# A Method for Automatically Predicting the Radiation-Induced Vulnerability of Unit Integrated Circuits

**DOI:** 10.3390/mi15040541

**Published:** 2024-04-18

**Authors:** Rui Dong, Hongliang Lu, Caozhen Yang, Yutao Zhang, Ruxue Yao, Yujian Wang, Yuming Zhang

**Affiliations:** Key Laboratory for Wide Band Gap Semiconductor Materials and Devices of Education Ministry, School of Microelectronics, Xidian University, Xi’an 710071, China; dongrui991024@163.com (R.D.); ycz1502961@163.com (C.Y.); ytzhang_xd@163.com (Y.Z.); yaoruxue4305@126.com (R.Y.); 15664600659@163.com (Y.W.); zhangym@xidian.edu.cn (Y.Z.)

**Keywords:** reliability, single event effect, soft error, TCAD

## Abstract

With the rapid development of semiconductor technology, the reduction in device operating voltage and threshold voltage has made integrated circuits more susceptible to the effects of particle radiation. Moreover, as process sizes decrease, the impact of charge sharing effects becomes increasingly severe, with soft errors caused by single event effects becoming one of the main causes of circuit failures. Therefore, the study of sensitivity evaluation methods for integrated circuits is of great significance for promoting the optimization of integrated circuit design, improving single event effect experimental methods, and enhancing the irradiation reliability of integrated circuits. In this paper, we first established a device model for the charge sharing effect and simulated it under reasonable conditions. Based on the simulation results, we then built a neural network model to predict the charge amounts in primary and secondary devices. We also propose a comprehensive automated method for calculating soft errors in unit circuits and validated it through TCAD simulations, achieving an error margin of 2.8–4.3%. This demonstrated the accuracy and effectiveness of the method we propose.

## 1. Introduction

**Figure 1:**
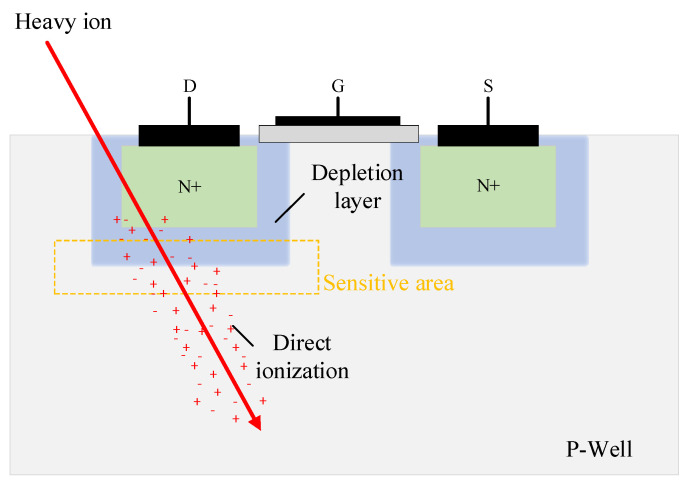
Schematic diagram of deposited charge generation through direct ionization by single particle incidence. The charge generated by ionization distributes along the trajectory of the particle.

Radiation particles from the space radiation environment have a severe impact on aerospace integrated circuits, with the radiation effects they receive primarily being single event effects (SEE) [1,2,3] and total ionizing dose effects (TID) [4,5,6]. The single event effects are illustrated in Figure 1. When high-energy particles penetrate semiconductor devices, they generate free charges along their path of incidence. As the penetration depth increases, the energy carried by the particles gradually decreases until it is depleted entirely, stopping within the semiconductor device. The charges generated by ionization are absorbed by the sensitive drain region, thereby forming a pulse current that leads to circuit functional disorder. With the continual reduction in device size and increase in layout density, the range of energy deposited by high-energy particles can cover multiple devices, significantly increasing the probability of multi-node charge collection between adjacent units (i.e., charge sharing effect), leading to single particle multiple transient phenomena. Furthermore, the reduction in device size also causes a decrease in critical charge and threshold for flipping, meaning that particles not directly hitting the sensitive areas of devices can also cause operational errors. Therefore, single event effects are becoming to be increasingly serious impacts on aerospace integrated circuits [7]. Studies have shown that single event effects are the main cause of failures in spacecraft today [8,9]. Hence, the evaluation of the radiation-induced vulnerability of integrated circuits is of great importance for promoting the design optimization of aerospace integrated circuits, improving experimental methods for single event effects, and enhancing the comprehensiveness of aerospace integrated circuit evaluations.

When evaluating large-scale processes, it is sufficient to consider only the netlist of the circuit. The netlist can only reflect the connection relationship of transistors, namely the topological structure of the unit circuit. However, as the process size continues to decrease, transistors become closer together and the probability of multiple transistors collecting charge at the same time increases, so, it is necessary to consider the actual physical layout to identify the adjacent transistor of the attacked transistor. The method of considering layout has become the mainstream trend of evaluation of the radiation-induced vulnerability of integrated circuits [10,11]. Among the currently proposed methods for assessing the single event effects in circuits, many are based on the Monte Carlo method [11,12,13,14]. However, it requires complex modeling and simulation, and greatly increases the consumption of computing resources to achieve good accuracy [15,16]. Therefore, developing more efficient methods for evaluating the sensitivity of integrated circuits is of significant importance.

This paper presents a method for automatically predicting the radiation-induced vulnerability of circuits. Initially, device modeling was performed using TCAD software (2018) to simulate the charge sharing effect. Based on simulation data, a relevant neural network model was established. Then, the Python (3.9) programming language was used to extract and parse relevant layout files and netlist information. This was followed by calling a SPICE simulator to perform fault injection simulation on each sensitive node under different input states. The soft errors of the unit integrated circuit were calculated through the flipping cross-section and the heavy ion differential LET spectrum, achieving a fully automated process. Finally, the results were validated, thereby demonstrating the accuracy of the proposed method for assessing the radiation-induced vulnerability of integrated circuits. Additionally, Python was used because it is a widely utilized programming language at present. Python has a vast user base, with many code developers providing excellent software packages for open access. The method proposed in this paper is built on Python and its numerous libraries.

The structure of this paper is as follows. Section 2 describes the process of device modeling and simulation using TCAD software. Section 3 establishes a neural network model to predict charge quantities based on simulation results. Section 4 describes our fully automated computation method, and finally, validation work is conducted in Section 5. Section 6 provides a summary of the paper.

## 2. Device Modeling and Simulation of Charge Sharing Effect

TCAD simulation is an advanced semiconductor device and process simulation software that utilizes finite element methods to solve semiconductor device characteristics and process-related issues. We utilized this software to conduct device characteristic simulations and radiation particle simulations for the processes under study.

**Figure 2:**
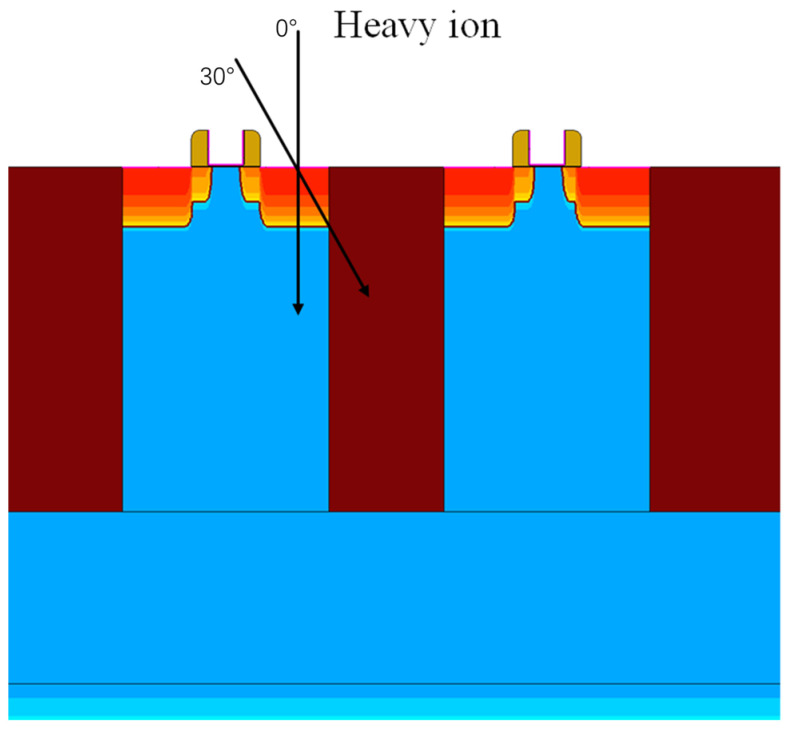
Single particle incidence simulation for primary and secondary devices. The left side represents the primary device, while the right side represents the secondary device. The black arrows represent the direction of particle incidence, with vertical indicating 0°.

A simulation model of a 28 nm bulk silicon CMOS process device was established using TCAD software. Considering that the charge sharing effect mainly occurs between two devices within the same well [17], taking NMOS as an example (the same applies to PMOS), the simulation schematic of the established three-dimensional device proximity arrangement model can be established as in Figure 2. It contains two NMOS devices. The black arrows represent the trajectory of the incoming particles. To mitigate the issue of increased gate leakage current due to reduced feature sizes, the device’s gate oxide layer employs a high dielectric constant material composed of HfO_2_ and SiO_2_. Furthermore, to further enhance the device’s performance, technologies such as lightly doped drain (LDD) and shallow trench isolation (STI) were utilized. The physical dimensions of the device are defined according to the minimum width-to-length ratio rule provided by the SPICE model, that is, a channel length of 30 nm, a channel width of 100 nm, and source and drain region lengths of 75 nm. When establishing the physical model of the device, various factors affecting the internal carrier distribution and mobility within the device must be considered. Based on the characteristics and structure of the 28 nm bulk silicon CMOS device, when the gate oxide thickness is small, quantum effects can cause changes in the gate capacitance and threshold voltage. Therefore, it is necessary to consider this influence when modeling. Additionally, the use of high-K materials at the HfO_2_ and SiO_2_ dioxide interface may lead to mobility degradation, thereby affecting the device’s conductive current. These influencing factors need to be considered during simulation to ensure the accuracy and reliability of the model. Based on the above principles, the following physical models were set: Fermi–Dirac statistics, high field saturation model, bandgap narrowing effect model, gate oxide quantum effect model, Shockley-Read-Hall (SRH) recombination, and Auger recombination model. The centroid of grid partitioning near the channel region can be densified by setting the minimum step size to 2 μm, and non-critical regions are partitioned using a less dense grid, enhancing the precision and convergence ease of the computational results. Models and grid partitioning strategy are applied to all TCAD simulations.

**Figure 3:**
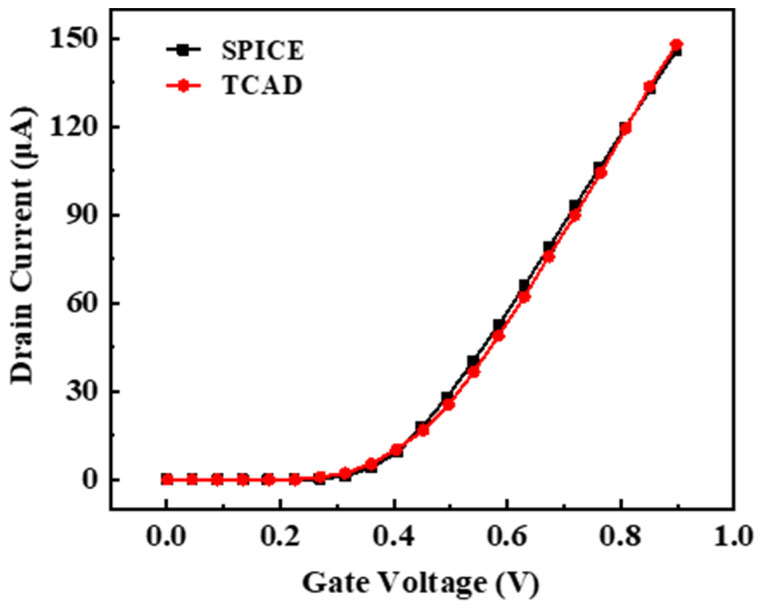
NMOS transfer characteristic curve calibration results. Twenty data points were selected from the range of 0 to 0.9 V.

To ensure the accuracy of the developed device model, simulations of the current-voltage characteristics were conducted on the constructed device model. These simulations were compared with the current–voltage characteristic curves in the 28 nm SPICE model. Based on the comparison results, the structural parameters of the device, such as doping concentration and junction depth, were continuously adjusted. Simulations were iteratively performed until the current–voltage curves of the device model closely matched those simulated by the SPICE model, thereby ensuring their electrical characteristics remained closely aligned. The results of this matching process are illustrated in Figure 3, which includes 20 simulation data points extracted from 0 to 0.9 V. It should be noted that the circuit-level simulation work in this paper was carried out by invoking the simulation software, which is a widely used circuit simulation tool in the industry, capable of accurately simulating the behavior of complex circuits.

We selected heavy ions as the type of incident particles for simulation. The device directly hit by the heavy ion is referred to as the primary device (the device on the left side corresponds to Figure 2), while the one passively collecting charges is called the secondary device (the device on the right side corresponds to Figure 2). Both are located in the same well and isolated by the STI layer, with two adjacent electrode regions being the devices’ drain regions. The initial state is set with the PN junctions of the two transistors reverse-biased, thus both transistors are in the off state, making their drain regions sensitive areas. The heavy ion’s incident point is centered in the transistor’s drain region. The penetration depth is set to 2 μm, and the track radius is 30 nm. During the simulation, the device spacing (D) was set to 30, 50, 100, 200, 300, 400, 500 nm, and the linear energy transfer (LET) of heavy ions was set to 0.1, 1, 10, 20, 40, 60, 80, 100 MeV·cm^2^/mg. The incidence angles of heavy ions were set to 0°, 15°, 30°, 45°, 60°, 75° (with 0° being perpendicular incidence on the main device, and the angle increasing as the incidence direction gradually shifts towards the secondary device, as shown in Figure 2). Simulations were conducted at these 336 ‘simulation points’ to obtain the charge absorbed in the drain region of the primary and secondary devices.

## 3. Establishment of the Single-Event Transient Pulse Source Model

The previous section provided a foundation by obtaining data through device-level simulations, which guided the establishment of a fault pulse model. In this section, a back propagation (BP) neural network model was constructed based on the simulation data to predict the charge amounts of primary and secondary devices. Thus, even for points falling between simulation points, it is possible to obtain the charge amounts of primary and secondary devices under complex conditions, which can then be used for pulse injection in subsequent automated evaluation processes.

The BP neural network is a type of multilayer feedforward neural network, characterized by signal forward propagation and error back propagation [18]. Its structure mainly consists of input layers, hidden layers, and output layers. The network performance is significantly influenced by the number of neurons and layers: too many neurons and layers can increase computational complexity, thereby slowing down the training speed; while too few neurons and layers may lead to insufficient network learning, affecting prediction accuracy. Therefore, selecting an appropriate number of neurons and layers is crucial.

To evaluate the accuracy of the BP neural network predictions, this paper selects R^2^ and RMSE as model evaluation metrics: (1) R^2^: Also known as the coefficient of determination, it reflects the model’s ability to explain the variability of the data. The closer its value is to 1, the higher the model’s fit to the data. (2) RMSE: Root mean square error measures the magnitude of differences between predicted values and actual values, quantifying the accuracy of predictions. The smaller its value, the higher the accuracy.

**Figure 4:**
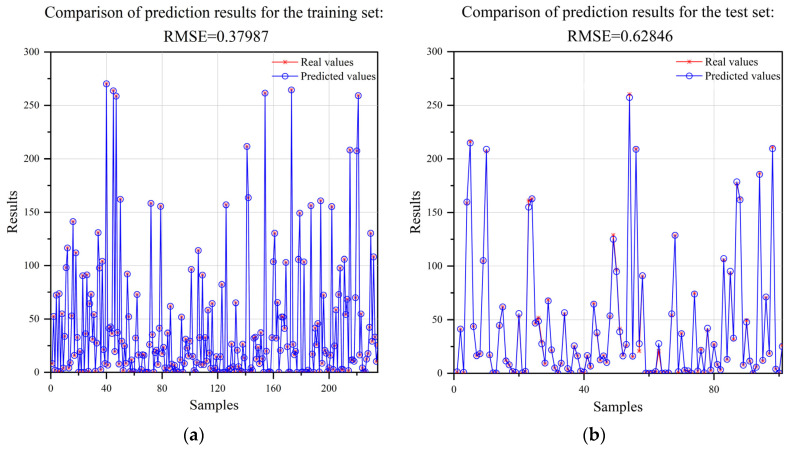

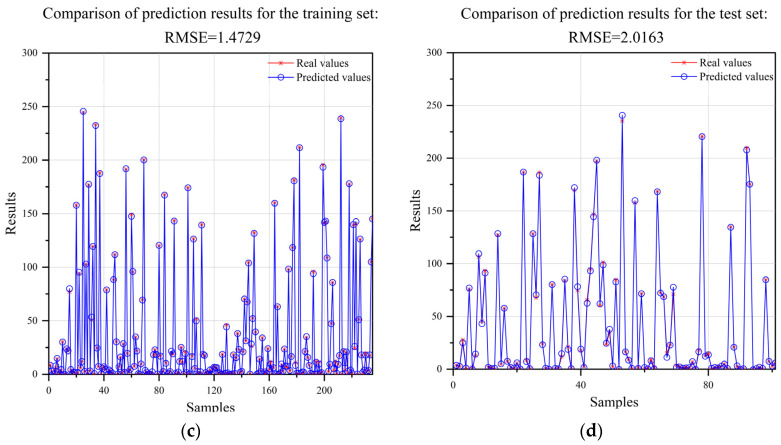
BP neural network model for predicting the charge amounts of primary and secondary devices. (**a**) Comparison of primary device charge amount predictions with the training set. (**b**) Comparison of primary device charge amount predictions with the test set. (**c**) Comparison of secondary device charge amount predictions with the training set. (**d**) Comparison of secondary device charge amount predictions with the test set.

After continuous optimization and adjustment, the neural network’s hidden layers are set to 2, with the first layer including 8 neurons and using the tanh activation function, and the second layer including 18 neurons with the sigmoid activation function. The iteration number is set to 1000, with a learning rate of 0.005. The R^2^ value is an important measure for determining the quality of a neural network, also known as the coefficient of determination. It is a key indicator of the performance of a regression model. The closer it is to 1, the better the model’s predictive ability. The R^2^ values for the training and testing sets of the primary device charge prediction model are 0.99982 and 0.99936, respectively. For the secondary device charge prediction model, the R^2^ values for the training and testing sets are 0.99377 and 0.98924, respectively. The neural network prediction results are shown in Figure 4a–d and represent the comparison of predictions for the training and testing sets of the primary and secondary device, respectively. The horizontal axis represents different samples, while the vertical axis corresponds to the charge amount predicted by the neural network model.

From an overall perspective, our model exhibits high accuracy in predicting charge quantities, with R^2^ approaching 1 and RMSE being below 2, as shown in Figure 4. This indicates that our neural network model effectively captures the complex relationship between input features and outputs, providing a reliable tool for predicting charge quantities.

**Figure 5:**
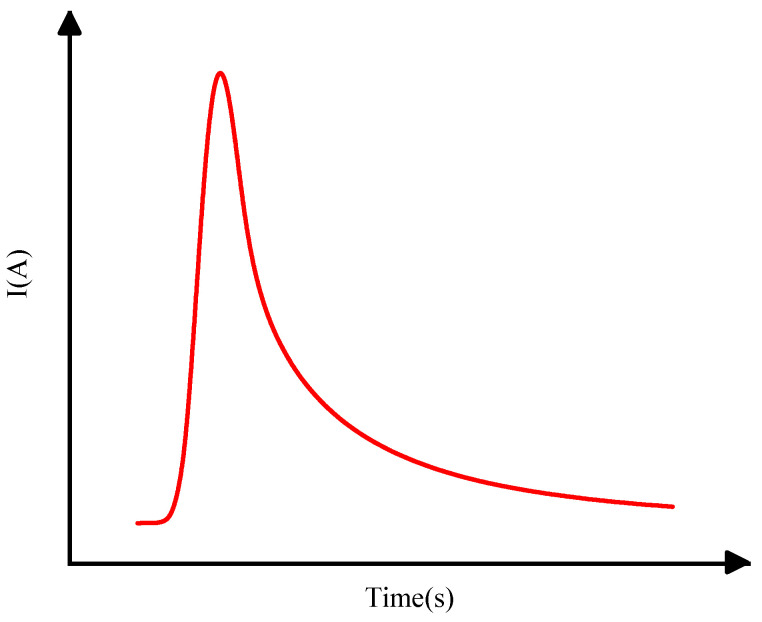
Double-exponential current source model waveform. With time on the horizontal axis, the current exhibits a steep rising edge and a gentle falling edge on the vertical axis.

The single-event current source model used in this paper is the double-exponential current source model [19], which can accurately simulate the bombardment of sensitive nodes in circuits by high-energy particles, making it suitable for circuit-level simulation methods [20]. Many outstanding works in the field of single-particle research are based on this model. It features a steep rising edge and a gentle falling edge (as shown in Figure 5).

Different amounts of charge collection correspond to pulse waveforms of varying sizes, but the basic shape and trend remain largely unchanged. The analytical formula is as follows:

(1) $$I(t)=I_{o}(e^{-\frac{t}{\alpha }}-e^{-\frac{t}{\beta }})$$

(2) $$I_{o}=\frac{Q_{\mathrm{tot}}}{\alpha -\beta }$$

where *I*_o_ is the peak transient current pulse, also a function of carrier mobility and the particle’s linear energy transfer (LET). *α* is the time constant for the circuit’s charge collection, *β* is the initial time of charge generation, and both *α* and *β* mainly depend on the device’s process characteristics such as electron mobility and donor density [21,22], obtained through simulation data of models established in the process library extracted from TCAD. This was conducted to improve the efficiency of modeling and subsequent calculations. *Q*_tot_ is the charge ionized by high-energy particles incident on the device, which is the main factor determining the double-exponential pulse. We predicted it under different incident conditions using the neural network model established earlier.

## 4. Automated Prediction Process for Radiation-Induced Vulnerability

**Figure 6:**
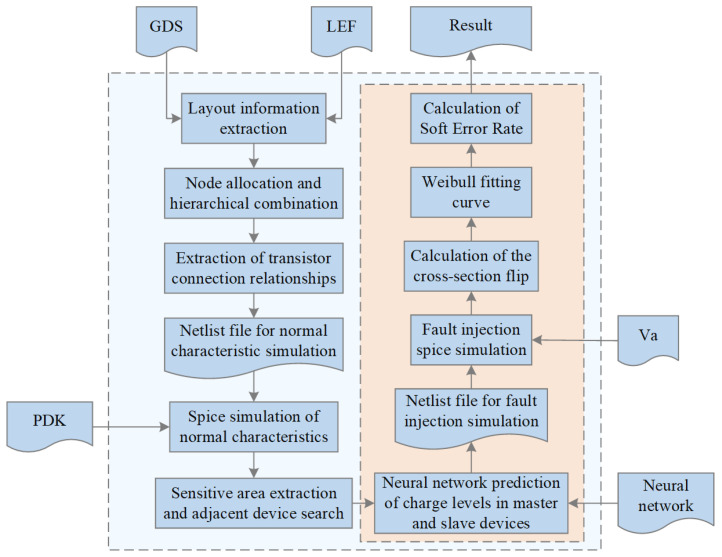
Automated evaluation method for integrated circuit sensitivity. The blue box inside the dashed line represents the main modules within the tool, while the blue box outside the dashed line represents the files or models that need to be called.

The framework of the integrated circuit sensitivity automatic evaluation method proposed in this paper is shown in Figure 6. Inside the larger dashed box in Figure 6 is the main part of the automated program, while outside this dashed box are the specific technical files and model files required by the process.

**Figure 7:**
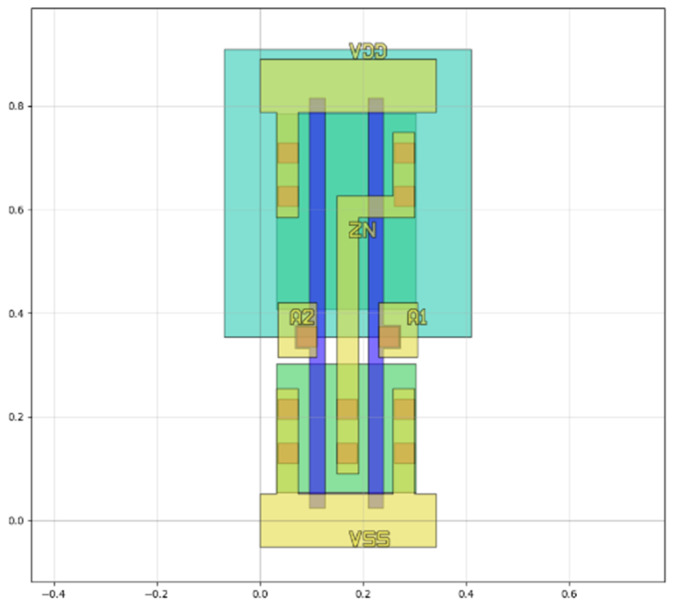
Extracted layout schematic (NOR). Blue represents the gate, green represents the source region, yellow represents the metal layer, and orange represents the contact hole. The unit is micrometers.

**Figure 8:**
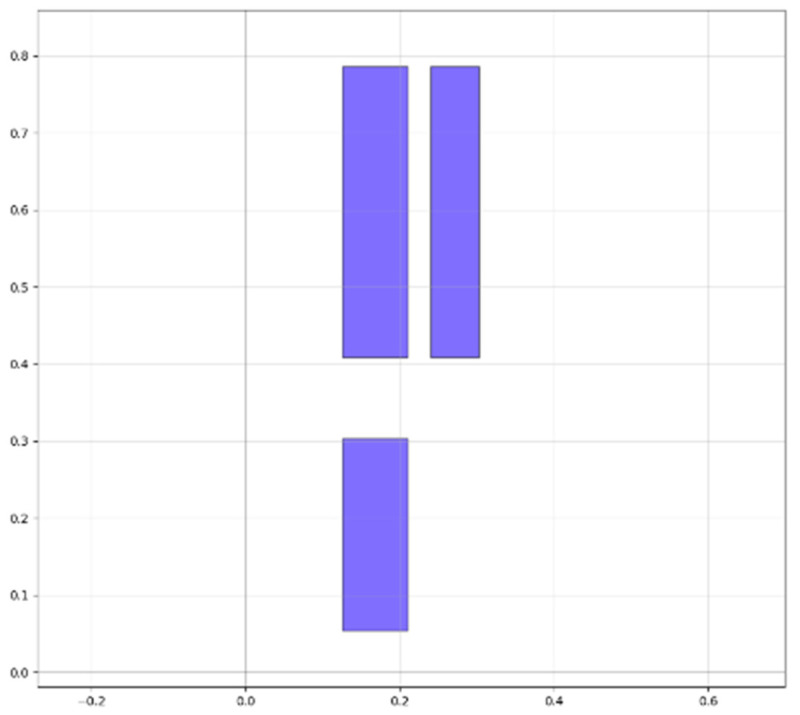
Schematic of the extracted sensitive area (NOR), mainly the drain region. The unit is micrometers.

When evaluating the 28-nm standard cell circuit, the gds and lef layout files are first processed to extract layers such as the active area, metal, N-well, contact, and polysilicon based on the layout’s coordinate and hierarchical information. This allows for the extraction of all their geometric information. Next, the input and output pins of the circuit are matched. Finally, by combining and matching different layers, we identify the positions and node names of the source, gate, and drain in the active area. Based on this, the transistor connection relations of the circuit can be extracted, further obtaining the netlist file used for normal state characteristic simulation. After calling the SPICE simulator for circuit-level steady-state characteristic simulation, the simulation results enable the extraction of the drain regions of off-state sensitive transistors under different input conditions. Figure 7 shows the layout schematic extracted by our method (taking NOR as an example); blue represents the gate, green represents the source region, yellow represents the metal layer, and orange represents the contact hole. According to the cross-checking of the netlist, circuit, and layout, it is evident that our developed tool can effectively extract the hierarchical structure of the layout and process it accordingly to further obtain relevant information. Figure 8 illustrates the sensitive area extracted by our method (also using NOR as an example), which is mainly the drain region. The units for the scales in both figures are in micrometers.

**Figure 9:**
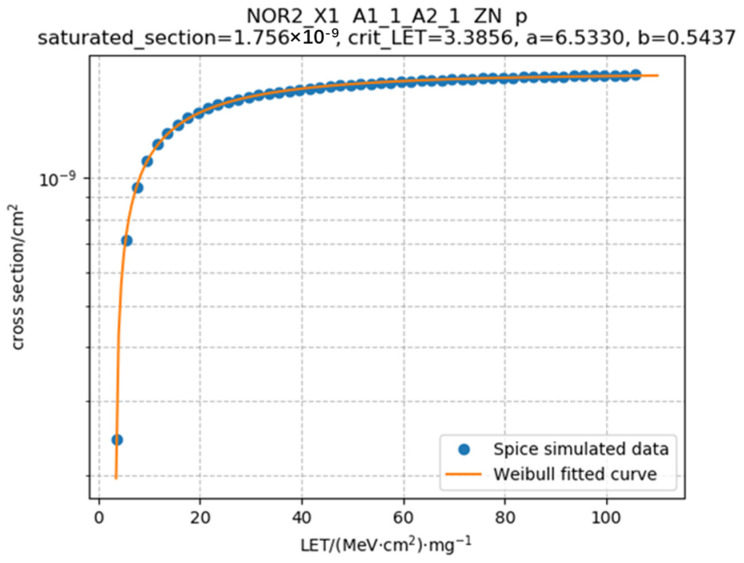
Schematic diagram of Weibull fitting effect. The blue dots represent the data points obtained from simulation, while the orange curve is the fitted Weibull curve.

Next, transistors near the sensitive transistor are searched for, and the previously trained neural network used to predict the charge quantity of the primary and secondary devices at specific angles and device spacings under different LETs, where angles are pre-set, and device spacings are based on extracted layout information. Subsequently, the pulse source file Va (a transient current source model written in Verilog-a language) is called, and by inputting the predicted charge quantity into the double-exponential function, fault pulse injection can be simultaneously performed on both primary and secondary devices, reflecting the charge sharing effect at the circuit level. This allows for the generation of a netlist file for fault injection simulation, conducting fault injection simulation to determine whether the output value of the unit circuit crosses the critical value VDD/2 (using VDD/2 as a uniform criterion for the evaluation method and subsequent result validation). Then, dichotomy search is used to continuously perform fault injection simulations until the critical condition is reached, where the LET value under that condition is the critical LET value for specific transistors connected to specific sensitive nodes under specific input states. With the critical LET value, the corresponding flipping cross-section can be obtained, and then automatic fitting of the LET value-flipping cross-section pairs with the Weibull curve is performed. The effect is as shown in the following Figure 9. Simulation yields several sets of data points, which are fitted to generate the orange curve.

The expression for the Weibull curve fitting is as follows:

(3) $$\sigma (LET)=\sigma_{\mathrm{sat}\;}\left\{1-\exp\left[-{\left(\frac{LET-LET_{\mathrm{th}\;}}{a}\right)}^{b}\right]\right\}$$

where σ(LET) is the saturation flipping cross-section, i.e., the value in the Weibull curve where the flipping cross-section no longer increases with *LET*, *LET*_th_ is the *LET* threshold, and *a* and *b* are shape fitting parameters.

The saturation cross-section values calculated in this paper are roughly on the order of 10^−10^ to 10^−8^ cm^2^, which is consistent with the research results from the relevant literature [23]. This, to some extent, confirms the accuracy of the method proposed in this paper. Our method initially obtains several sets of LET value-flipping cross-section pairs, which are then fitted to a Weibull curve to derive the final curve results. Obtaining the final results typically requires several tens of seconds. In contrast, referencing Monte Carlo methods, their extensive random simulations and computational complexity limit CPU time, which may persist for several hours or even days [16]. Therefore, in comparison, our method offers faster computational speed, enabling rapid assessment of the circuit under test.

**Figure 10:**
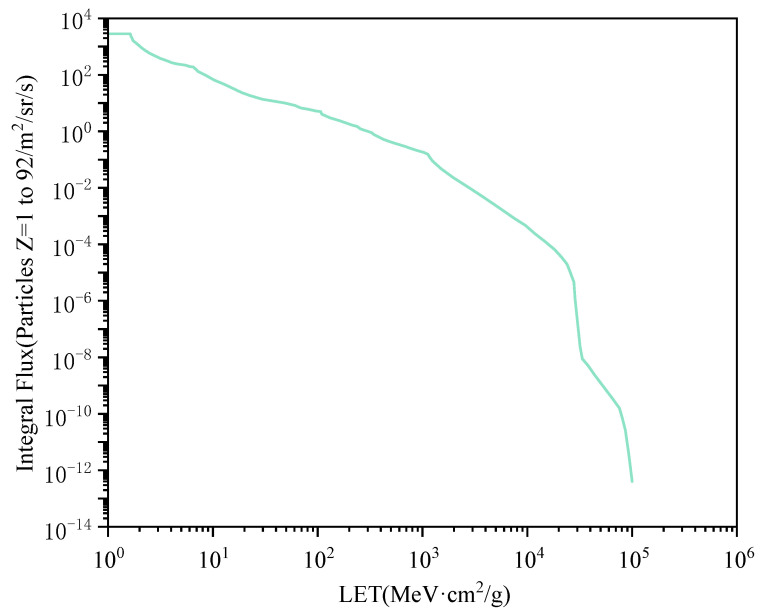
Heavy ion differential LET spectrum [24].

A reasonable heavy ion differential LET spectrum is selected, with this paper adopting the relationship between the heavy ion differential flux and the LET value in the Si material during the minimum solar activity year in geosynchronous orbit given by CRÈME [24], shown in Figure 10.

Multiplying the heavy ion differential LET spectrum curve by its expression, selecting a reasonable upper and lower limit, and integrating, the corresponding SER value can be obtained, with the following integration expression:

(4) $$SER=\int_{LET_{\min}}^{LET_{\max}}\phi (LET)\sigma (LET)dLET$$

where ϕ(LET) is the heavy ion differential LET spectrum, σ(LET) is the flipping cross-section curve expression, and *SER* is the soft error rate value of the sensitive tube connected to a specific sensitive node under a specific state, in units of FIT.

The smaller dashed box on the right side of Figure 6 indicates that the internal process needs to be repeated. For each unit, there will be multiple different input states, traversing each sensitive node under each input state and each sensitive transistor connected to that node. Summing the *SER* for each case yields the total *SER* value for the corresponding unit circuit.

## 5. Result Verification

In this section, we validate the method proposed in this paper. The validation in this section is based on the 3D-TCAD modeling and heavy ion irradiation simulation of the unit circuit implemented in the previous modeling process, aiming to simulate the response of the real unit circuit under radiation. This provides an important reference standard for validating the results of the method proposed in this paper.

**Figure 11:**
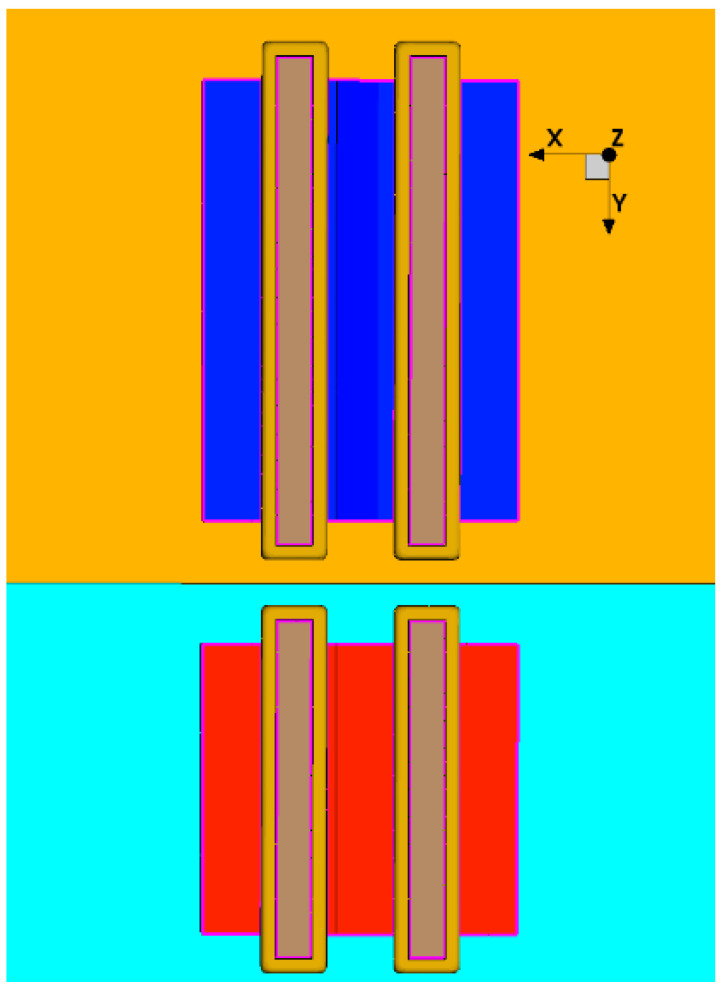
NOR circuit constructed in TCAD, including two NMOS (red area below) and two PMOS (blue area above).

**Figure 12:**
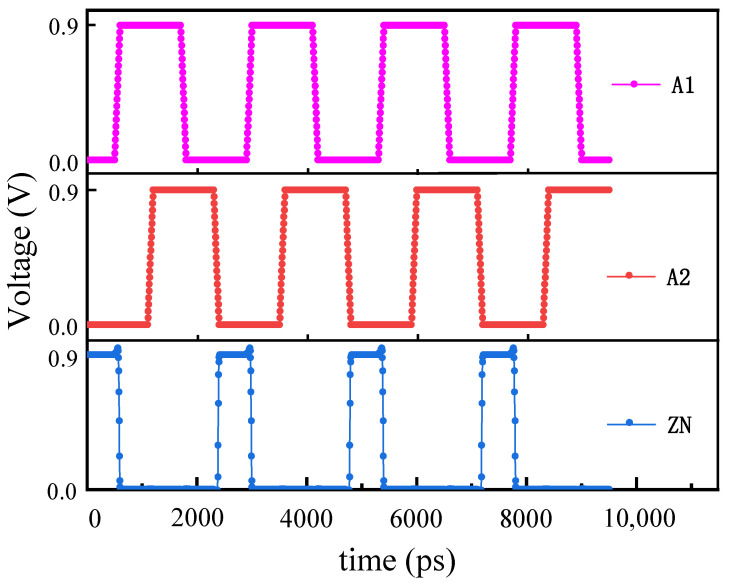
Waveform diagram of the NOR gate’s node signals. A1 and A2 are the input pins of the NOR gate, and ZN is the output pin.

The steps involve constructing the corresponding circuit in TCAD software, taking NOR as an example. The constructed model is as shown in Figure 11 and includes two NMOS and two PMOS with dimensions and location information derived from the gds file of the corresponding standard cell circuit. Then, we perform steady-state characteristic simulation on it and apply appropriate bias voltages to the input ports to ensure correct outputs under all conditions (as shown in Figure 12, which displays the TCAD simulation curves of an NOR with about 2000 simulation points). In Figure 12, A1 and A2 are the input pins of the NOR gate, and ZN is the output pin. The input pins have undergone several logic value changes, and the output pin can correspondingly output the correct results. This process demonstrates that the established unit circuit model can be used for subsequent single-particle incidence simulations.

**Figure 13:**
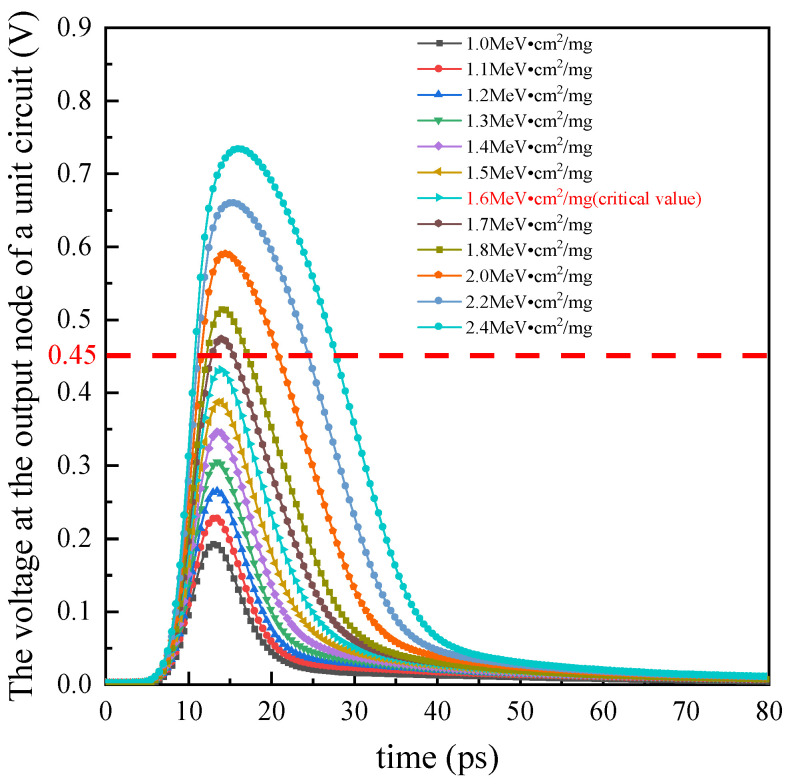
The output node voltage for a circuit unit subjected to single particle incidence in TCAD. The red dashed line represents the threshold voltage.

As shown in Figure 13, the output voltage curve is obtained by continuously changing the LET value of a single particle incident on the PMOS transistor connected to the NOR gate’s ZN node under the 01 state. With input terminals A1A2 set to 01, the output voltage should remain unchanged at 0. However, the injection of heavy ions will generate a single-event pulse at this time. From Figure 13, the output voltage, originally at 0, will increase momentarily at a certain instant, then return to a low voltage state. As the LET increases, the peak of the pulse increases, and the pulse width also increases. The red dashed line represents the threshold voltage, which is typically half of the power supply voltage, i.e., 0.45 V. It can be observed that the critical LET value is 1.6 MeV·cm^2^/mg under the input condition of 01, with subsequent calculations being the same as in the previous section.

Due to the high computational resources and time consumption of TCAD simulations, in this section, we only validate the scenario of particles incident vertically on the primary device. We also select the output results under vertical conditions and then compare these results to calculate the error. The related results are as follows in Table 1. It should be noted that in Table 1, “TCAD (golden)” refers to the results obtained from TCAD simulations, serving as the reference standard. All the SER values are expressed in FIT. It can be seen that the error in the SER values, calculated based on the results obtained from TCAD, is very small, essentially ranging between 2.8% and 4.3%. This demonstrates the effectiveness and accuracy of the method proposed in this paper.

## 6. Conclusions

In this paper, we first established a device model for the charge sharing effect, simulated it under reasonable conditions, and then built a neural network model to predict the charge amount in primary and secondary devices based on the simulation results. We propose a comprehensive automated method for calculating soft errors in unit circuits and validated it through TCAD simulations, achieving an error margin of 2.8–4.3%. This proved the accuracy and effectiveness of the method we propose.

## Figures and Tables

**Table 1:** Comparison of SER calculated by the method of this paper and the results from TCAD.

Logical Circuit Unit	TCAD (Golden) (FIT)	Method of This Paper (FIT)	Error
NAND	10.0	9.7	2.8%
NOR	12.8	12.3	4.3%
INV	3.8	3.7	3.1%
AND	21.2	20.5	3.6%
OR	19.6	18.9	3.4%

## Data Availability

The data presented in this study are available on request from the corresponding author. The data are not publicly available due to confidentiality of the project.
